# Author Correction: Construction of Pd-Zn dual sites to enhance the performance for ethanol electro-oxidation reaction

**DOI:** 10.1038/s41467-022-28541-z

**Published:** 2022-02-11

**Authors:** Yajun Qiu, Jian Zhang, Jing Jin, Jiaqiang Sun, Haolin Tang, Qingqing Chen, Zedong Zhang, Wenming Sun, Ge Meng, Qi Xu, Youqi Zhu, Aijuan Han, Lin Gu, Dingsheng Wang, Yadong Li

**Affiliations:** 1 Department of Chemistry, Tsinghua University, Beijing, China; 2 College of Chemistry and Materials Engineering, Wenzhou University, Wenzhou, Zhejiang China; 3 State Key Laboratory of Chemical Resource Engineering, Beijing University of Chemical Technology, Beijing, China; 4 State Key Laboratory of Coal Conversion, Institute of Coal Chemistry, Chinese Academy of Sciences, Taiyuan, Shanxi China; 5 State Key Laboratory of Advanced Technology for Materials Synthesis and Processing, Wuhan University of Technology, Wuhan, China; 6 Key Laboratory of Functional Molecular Solids, Ministry of Education, College of Chemistry and Materials Science, Anhui Normal University, Wuhu, Anhui China; 7 College of Science, China Agricultural University, Beijing, China; 8 Research Center of Materials Science, Beijing Institute of Technology, Beijing, China; 9 Institute of Physics, Chinese Academy of Sciences, Beijing, China

**Keywords:** Electrocatalysis, Electrocatalysis, Nanoparticles

Correction to: *Nature Communications* 10.1038/s41467-021-25600-9, published online 6 September 2021.

In this article the wrong figure appeared as Supplementary Fig. 23; the figure should have appeared as shown below. The original article has been corrected. The incorrect figure remains present in the Transparent Peer Review file. 

## Supplementary information

Updated Supplementary Information

