# Supplementary material for: Construction of Pd-Zn dual sites to enhance the performance for ethanol electro-oxidation reaction
Source: Nat Commun. 2021 Sep 6;12:5273. doi: 10.1038/s41467-021-25600-9 (PMC8421426; doi:10.1038/s41467-021-25600-9)
Supplement: Supplementary file 1 — Supplementary Information [file 41467_2021_25600_MOESM1_ESM.pdf]

Supplementary Information

Construction of Pd-Zn Dual Sites to Enhance the Performance for Ethanol Electro-Oxidation Reaction

Qiu et al.

Table of Contents

Table of Contents 2
Experimental Section 3
Chemicals 3
Synthesis of Pd_1/NC@ZnO 3
Synthesis of Pd_n/NC@ZnO 3
Synthesis of PdZn/NC 3
Synthesis of PdZn/NC@ZnO (5%) 3
Electrochemical Measurements 4
Product Analysis: 4
Membrane Electrode Assembly (MEA)： 5
Supplementary Figures and Tables 6
References. 28

Experimental Section

Chemicals

Zincacetate Dihydrate ($Zn(OAc)_2 \cdot 2H_2O$) and Sodium hydroxide (NaOH) were purchased from Xilong Scientific Co., Ltd. Dopamine hydrochloride (DA·HCl) was purchased from Beijing HWRK Chem Co. LTD. Tris(hydroxymethyl) aminomethane (Tris) was purchased from Alfa Aesar (China) Chemicals Co., Ltd. Urea, ethanol, isopropanol and deionized water was purchased from Beijing Tongguang Fine Chemicals Company. Commercial Pd/C catalyst (5 wt% Pd supported on carbon black) and Sodium tetrachloropalladate (II) (Na_2PdCl_4) was purchased from Innochem. Palladium(II) acetylacetonate ($Pd(acac)_2$) and Nafion solution (5%) were purchased from Sigma-Aldrich. All chemicals were of analytical grade and used as received without further purification.

Synthesis of Pd_1/NC@ZnO

100.0 mg ZnO nanorods were dispersed in 20 mL Tris buffer solution (10 mM, pH = 8.5) by ultrasonication labeled as solution I. Dopamine hydrochloride (100.0 mg) and palladium(II) acetylacetonate (1.4 mg, 0.5 wt.% to dopamine) were dissolved in a solution of ethanol (2 mL) and deionized water (3 mL), and labeled as solution II. Solution II was then injected into solution I under stirring. The resulting suspension was stirred continuously for 12 h at room temperature. The resulting $Pd(acac)_2$/PDA@ZnO nanorods were isolated by centrifugation and washed with water three times. Pd_1/NC@ZnO were obtained by pyrolyzing the $Pd(acac)_2$/PDA@ZnO nanorods at 200 °C in N_2 atmosphere for 2 h in a tube furnace at a ramp rate of 5 °C min^{-1}.

Synthesis of Pd_n/NC@ZnO

The Pd_n/NC@ZnO was obtained by a similar procedure to PdZn/NC@ZnO except that the $Pd(OH)_2$/ZnO@PDA was treated at 400 °C in N_2 atmosphere for 2 h in a tube furnace at a ramp rate of 5 °C min^{-1}.

Synthesis of PdZn/NC

The PdZn/NC@ZnO was washed in 5 M HCl for 2 h at room temperature. Finally, the resulting product PdZn/NC was obtained after the samples were centrifuged, washed with deionized water several times, and dried at 80 °C in an oven.

Synthesis of PdZn/NC@ZnO (5%)

When we prepared PdZn with high loading, the other conditions remained unchanged except that the Na_2PdCl_4 precursor was increased by five times. A sample with higher Pd loading (5%) is prepared using the same method.

Electrochemical Measurements

Electrochemical measurements were carried out with a three-electrode system on an electrochemical workstation (CHI 660, Shanghai Chenhua, China). Our experiments were performed with a saturated Ag/AgCl electrode (Ag/AgCl) electrode as the reference electrode. It was calibrated to *E* (RHE, reversible hydrogen electrode) from *E*(Ag/AgCl) by following the formula *E*(RHE) = *E*(Ag/AgCl) + 0.197 + 0.05916*pH* (1). 10 mg catalysts were dispersed in 950 μL isopropanol，950 μL deionized water and 100 μL of 0.5 wt% Nafion solution and then followed by an intense sonication of 2 h to get a homogeneous 5 mg mL^{-1} catalyst ink. After that, 20 μL inks (equal to 10 μg of catalyst) were deposited onto a 5 mm diameter glassy carbon rotating disk electrode (RDE, Pine Research Instrumentation) and dried to form a uniform thin film that was further used as a working electrode. Therefore, the actual Pd loadings of PdZn/NC@ZnO, Pd_1/NC@ZnO, Pd_n/NC@ZnO and commercial Pd/C are 0.03008, 0.03458, 0.03983 and 0.50000 μg, respectively. A carbon rod was used as the counter electrode, and a saturated Ag/AgCl electrode was used as the reference electrode. All of the CV measurements were obtained at room temperature. For CO stripping tests, CO oxidation experiments were carried out in the solution of 1.0 M KOH 20 mV s^{-1}. Before the test, the solution was purged with N_2 for 30 min and then was bubbled with CO gas (99.9%) for 15 min at 0.1 V to achieve the maximum coverage of CO at the Pd active centres. The residual CO in the solution was excluded by nitrogen for 30 min. The ECSA were obtained by integrating the charge of the COad layer involved in an anodic stripping peak between 0.10 V and 1.40 V vs RHE after the linear background correctting. The ECSA of Pd can be calculated based on the following equation:

$$\mathrm{ECSA_{CO}} = \frac{Q_{CO}}{m * q_{CO}} \qquad (2)$$

where Q_{CO} is the charge for the oxidation of CO (mC cm^{-2}), *m* is the loading amount of metal, and q_{CO} is the charge required to oxidize the monolayer of CO (420 μC cm^{-2}) on the catalyst.

Product Analysis:

The EOR was conducted in a gas-closed circulation system at room temperature. Specifically, a electrochemical cell was connected to a gas circulation system that was connected to a gas chromatography to analyze the products online every 1 h. The system was first flushed by He (20 ml min^{-1}, for 30 min) for three times before tests to remove the air. Then the 1.0 M KOH and 1.0 M ethanol electrolyte solution was saturated by He stream for 30 min. The working electrode was initially cycled between -0.85 and 0.35 V at 50.0 mV s^{-1} in 1.0 M KOH and 1.0 M ethanol for several cycles to remove the residual ligands on catalyst surface. And then, the chronoamperometry measurements were conducted at -0.30 V vs Ag/AgCl in the solution of 1.0 M KOH and 1.0 M ethanol. The evolved gaseous products were analyzed online by a gas chromatography (GC, Shimadzu, Tracera (GC-2010 Plus A, Barrier Discharge Ionization Detector (BID)-2010 Plus)) with a GC column (Shinwa Chemical Industries, Micropacked ST) and He carrier gas (purity no less than 99.999%). After the chronoamperometry measurements we analyzed the solution through Ion Chromatography and the ^{1}H NMR. Instrument configuration and chromatographic conditions: Ion Chromatography (ICS-1100, thermo Dionex) equipped with conductivity detector, AS-DV automatic sampler, protected and analytical column: AG19 (2*50 mm) and AS19 (4*250 mm)

analytical column, eluent KOH concentration of 20 mM, flow rate of 1 mL min^{-1}, inhibition current of 50 mA, column temperature of 30 °C. After the chronoamperometry measurements, the solution was collected directly. The sample was diluted 10 times with ultrapure water and then directly tested on the instrument (It has been tested directly on the instrument, but the matrix is complex and has interference). In order to further prove the nature of ethanol oxidation products, the solution was detected by ^1H NMR. After the current density experiments, the solution was treated by rotatory evaporation. The white colloidal substance obtained by rotary evaporation is dissolved by deuterium water.

Membrane Electrode Assembly (MEA):

Catalysts (0.2 g) were mixed with 5 mL deionized water under stirring. Then 2 mL Nafion DE 520 (5 wt% in isopropyl alcohol) solution was added to the mixture, followed by ultrasonic treatment for 30 min to form catalyst slurry. Then the catalyst slurry was applied to PTFE thin film by spraying, after dried at 60 °C for 10 min followed with at 90 °C in N_2 atmosphere for 3 min, the catalyst layer was then transferred onto the polymer electrolyte membrane (Nafion212, 50 μm) at 125 °C and 5 MPa by the decal method to form the catalyst-coated membrane (CCM). The GDL (Toray TGP-H-060) was placed on the anode and cathode side of the CCM to form the MEA with Active area of 2×2 cm^2. The cathode was prepared by the Pd/C catalyst with Pd loading of 0.05 mg cm^{-2} (Innochem, 5 wt% Pd on carbon).

Fuel:

The electrolyte containing 1.0 M ethanol and 1.0 M KOH was fed into the anode at the rate of 2.0 mL min^{-1}, cathode oxygen flow rate 100.0 sccm. The cell temperature is 50 °C.

Test System:

Fuel Cell Test System (Hephas 850e)

The Anode:

The anode was prepared by the as-synthesized catalysts with loading of 0.8 mg cm^{-2}. The anode was prepared by Pd/C catalyst with Pd loading of 1.0 mg cm^{-2} (Innochem, 5 wt% Pd on carbon).

Supplementary Figures and Tables

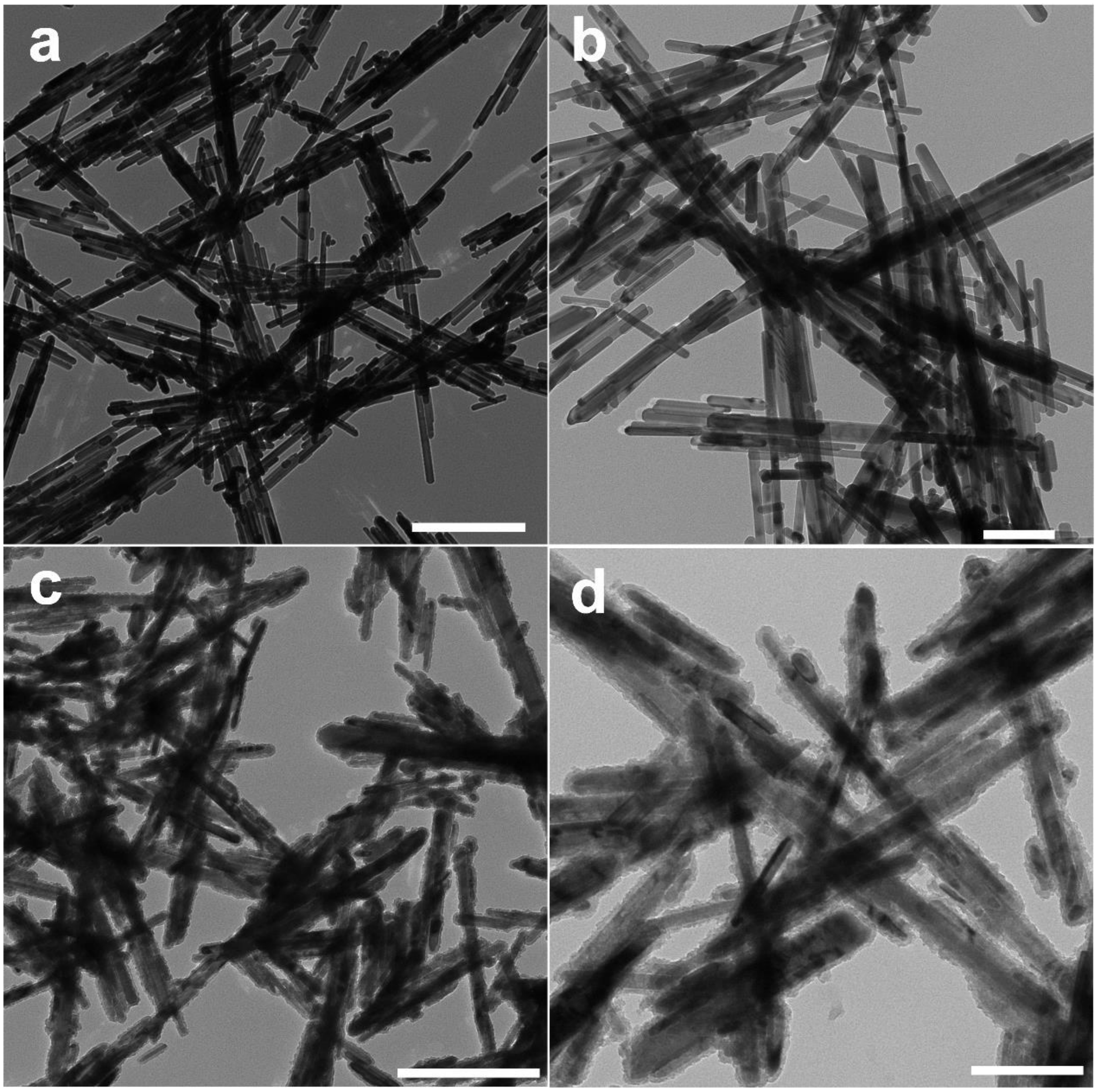

Supplementary Figure 1 | TEM images of ZnO and ZnO@PDA. a ZnO, Scale bar, 500 nm; **b** ZnO, Scale bar, 200 nm; **c** ZnO@PDA, Scale bar, 500 nm; **d** ZnO@PDA, Scale bar, 200 nm.

It can be clearly observed that ZnO, ZnO@PDA, and ZnO NRs are synthesized in uniform-sized distribution, PDA is coated on the out surface of ZnO NRs regularly.

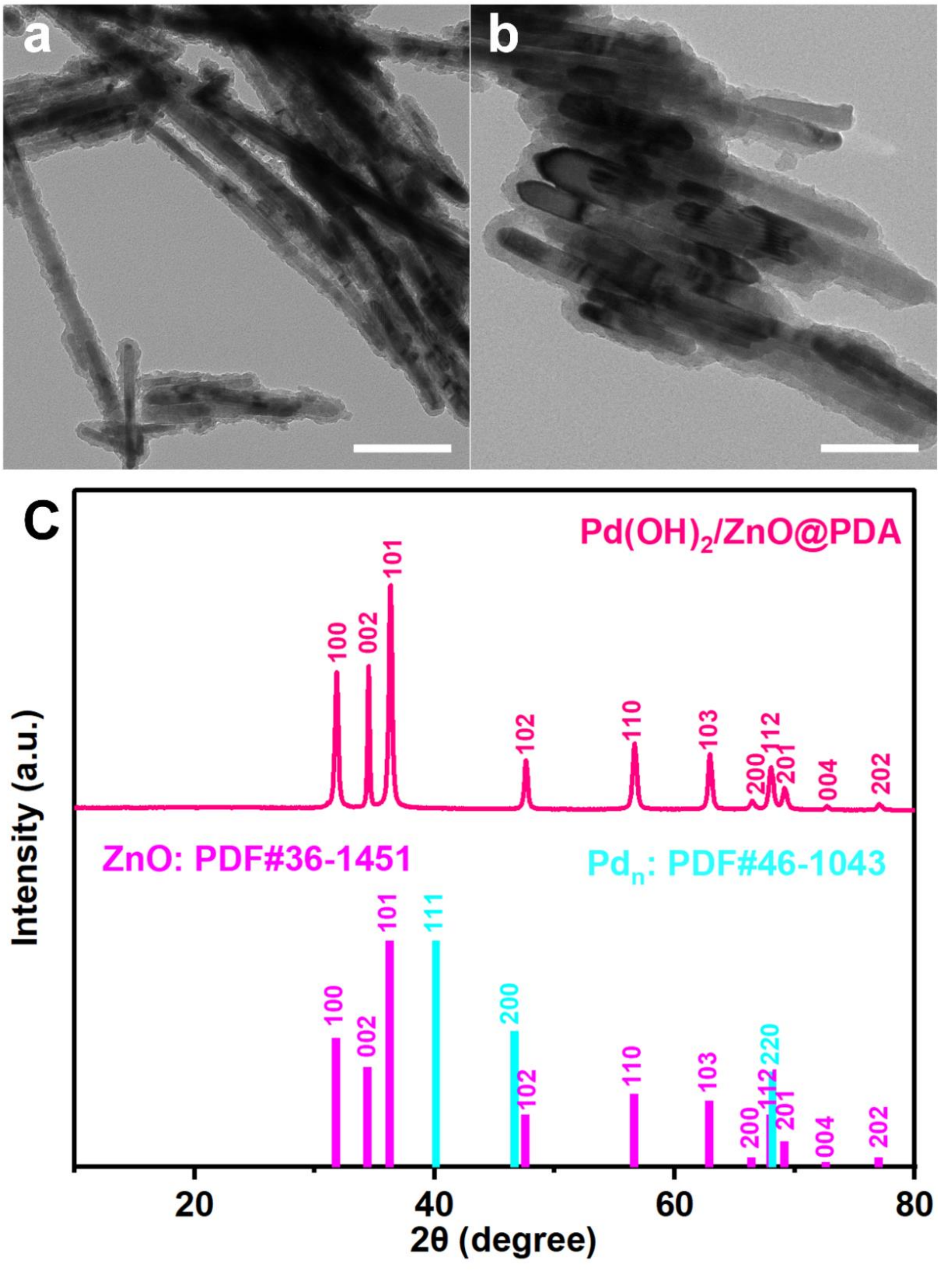

Supplementary Figure 2 | TEM images and XRD pattern of $Pd(OH)_2$/ZnO@PDA. **a** TEM image of $Pd(OH)_2$/ZnO@PDA, Scale bar, 200 nm; **b** TEM image of $Pd(OH)_2$/ZnO@PDA, Scale bar, 100 nm; **c** XRD pattern of $Pd(OH)_2$/ZnO@PDA.

As can be seen from TEM image of $Pd(OH)_2$/ZnO@PDA at different magnifications, no obvious nanoparticles can be observed on the PDA layer. The diffraction peaks for $Pd(OH)_2$/ZnO@PDA can match well with ZnO. No obvious peaks of Pd can be observed in the $Pd(OH)_2$/ZnO@PDA diffraction patterns due to the small size of $Pd(OH)_2$ nanoparticles.

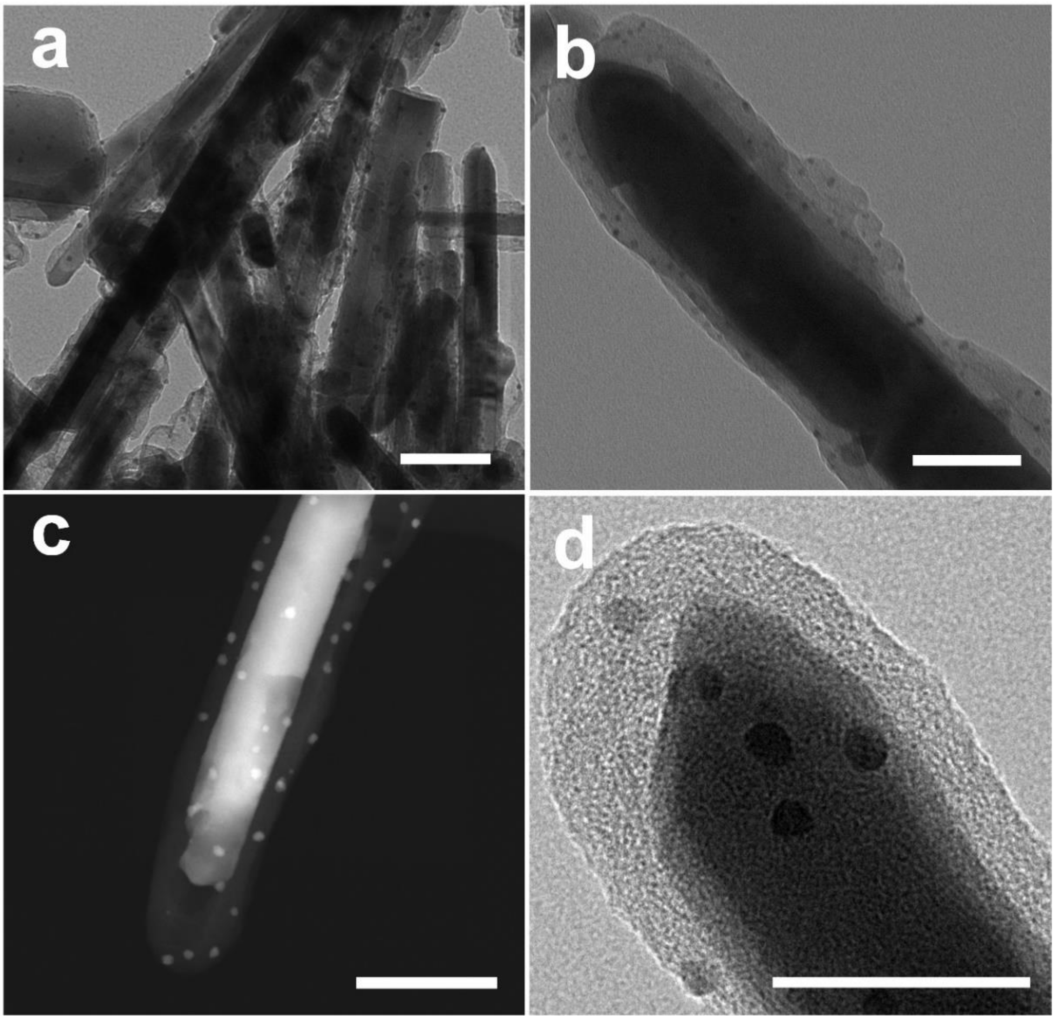

Supplementary Figure 3 | TEM, STEM and HR-TEM images of PdZn/NC@ZnO. **a**, **b** TEM images of PdZn/NC@ZnO, Scale bar, 100 nm; **c** STEM image of PdZn/NC@ZnO, Scale bar, 100 nm; **d** HR-TEM image of PdZn/NC@ZnO, Scale bar, 50 nm.

It can be clearly observed from TEM images of the as-obtained PdZn/NC@ZnO at different magnifications that PdZn NPs are uniformly distributed on the NC@ZnO carrier. STEM and HR-TEM images of PdZn/NC@ZnO also show obvious small PdZn NPs presented on the NC@ZnO.

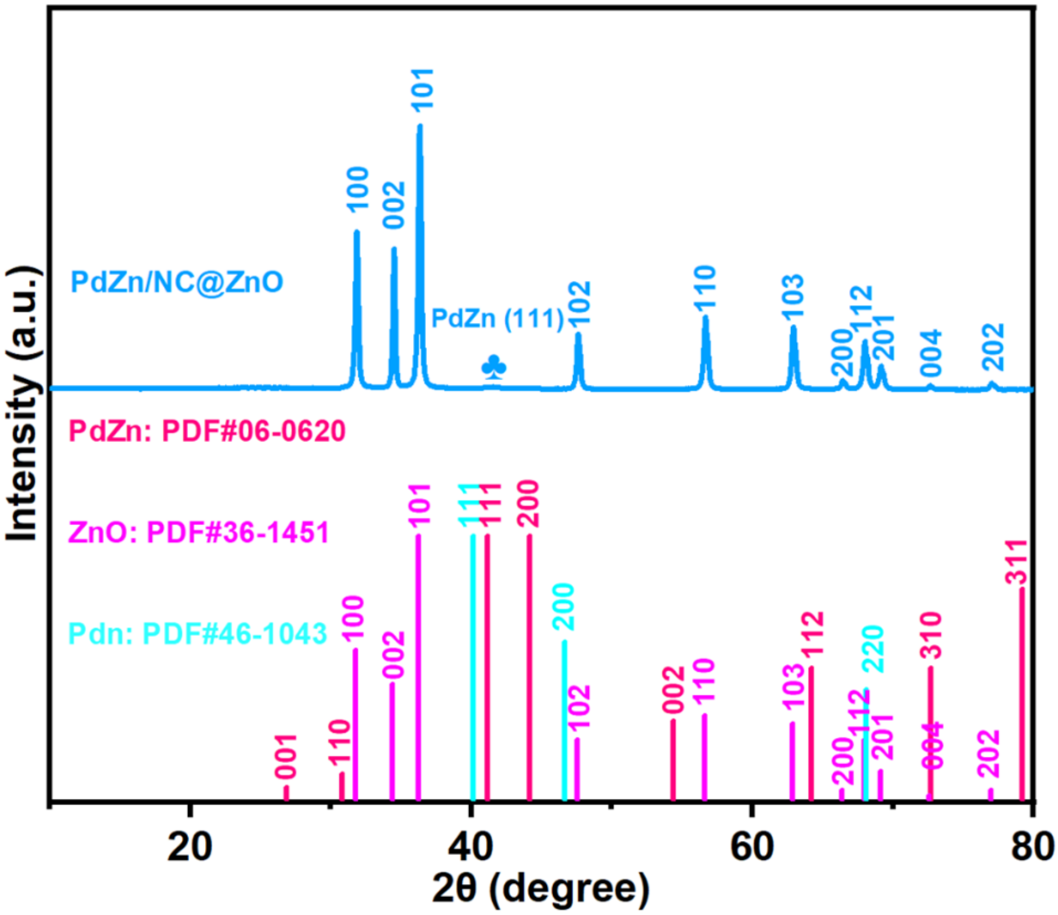

Supplementary Figure 4 | XRD pattern of PdZn/NC@ZnO.

The diffraction peaks for PdZn/NC@ZnO can match well with ZnO. No obvious peaks of PdZn NPs can be observed in the PdZn/NC@ZnO diffraction pattern due to their small size.

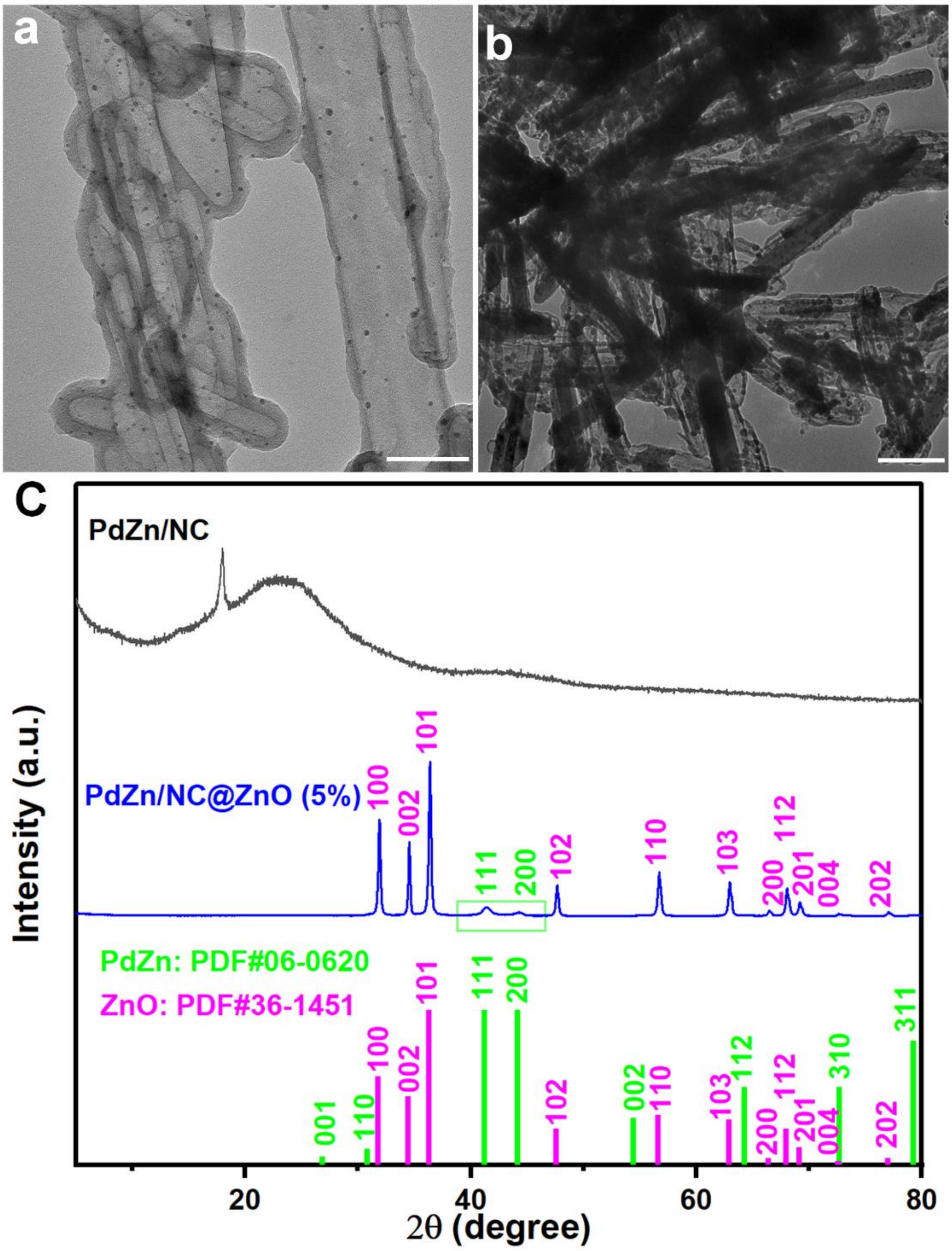

Supplementary Figure 5 | Characterizations of PdZn/NC and PdZn/NC@ZnO (5%). a TEM image of PdZn/NC. Scale bar, 100 nm. **b** TEM image of PdZn/NC@ZnO (5%) Scale bar, 200 nm. **c** XRD pattens of PdZn/NC (black line) and PdZn/NC@ZnO (5%) (blue line).

After the ZnO is washed away, there are still PdZn nanoparticles on the NC carrier in the Supplementary Fig. 5a and only graphitic carbon peaks were observed because of the ultrasmall size of the PdZn nanoparticles in the Supplementary Fig. 5c. When the loading of Pd precursor is increased five times, it can be seen from Supplementary Fig. 5b that the amount and size of PdZn NPs become more and larger a lot. For this sample, the featured intermetallic PdZn (PDF#06-0620) signal can be observed in the XRD pattern of PdZn/NC@ZnO (5%) (Supplementary Fig. 5c).

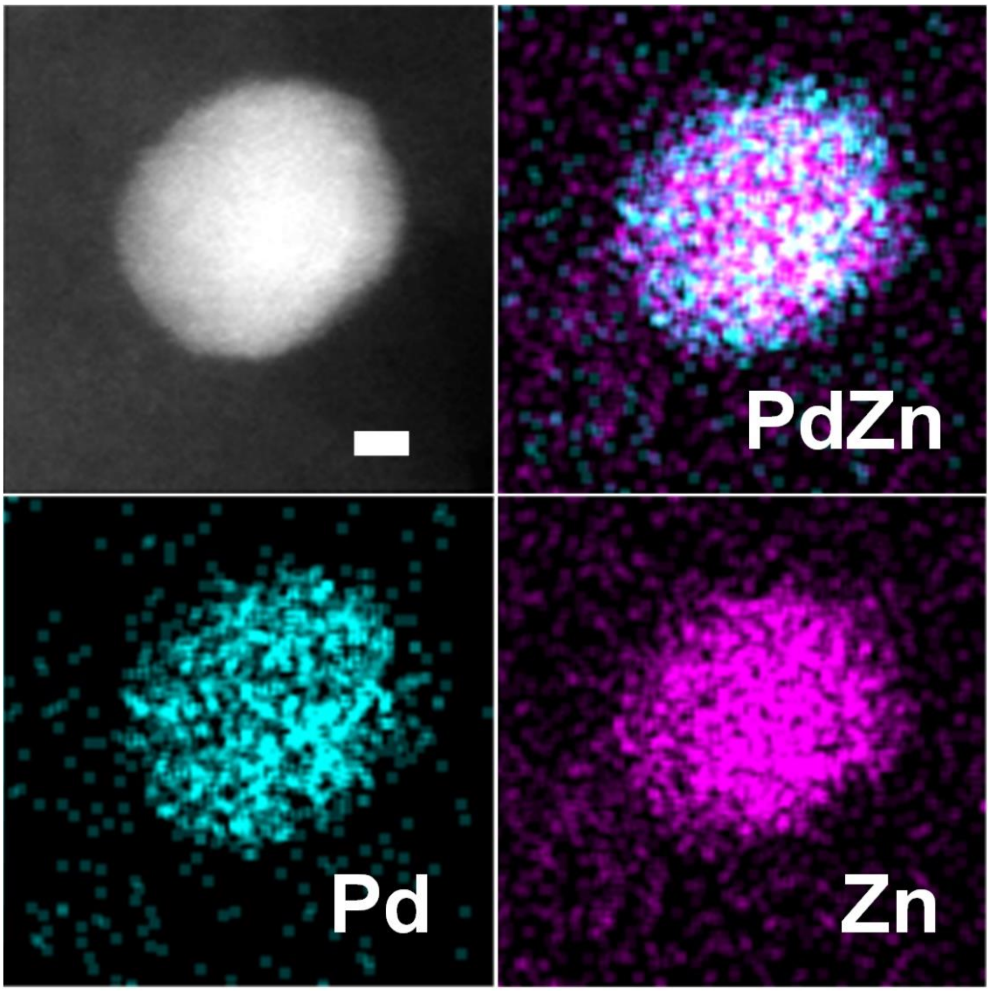

Supplementary Figure 6 | AC HAADF-STEM image and corresponding elemental mappings of a PdZn NP in PdZn/NC@ZnO. Scale bar, 2 nm.

It shows that a uniform dispersion of Pd and Zn on the PdZn NP.

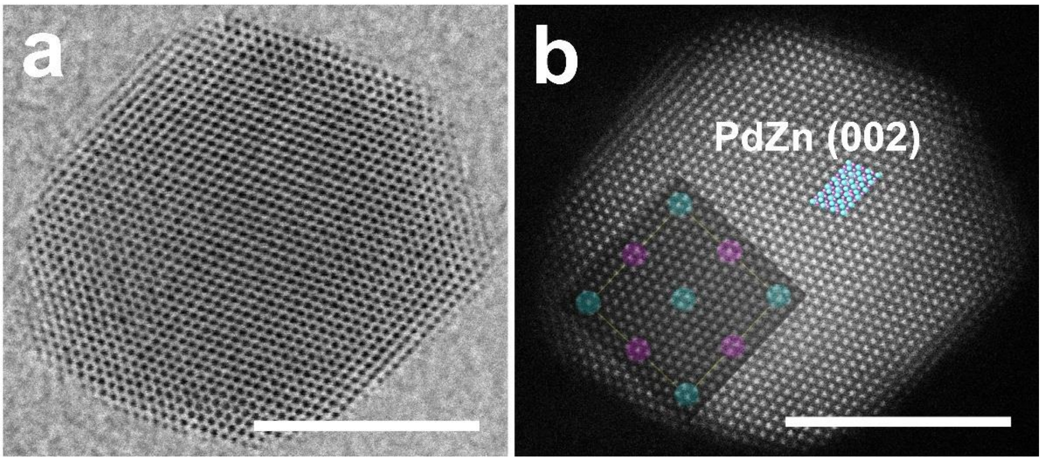

Supplementary Figure 7 | AC HAADF-STEM image of a PdZn NP. a AC HAADF-STEM image of a PdZn NP in bright field. Scale bar, 5 nm. **b** AC HAADF-STEM image of a PdZn NP in dark field, the insert is crystal structure of PdZn (002) Plane, Pd atoms (blue) and Zn atoms (purple). Scale bar, 5 nm.

It shows orderly distributed Pd-Zn dual sites in bright field. The atomic arrangement with the lattice spacing of 1.69 Å can match well with the interplane distance of (002) plane on the ordered PdZn intermetallic compound.

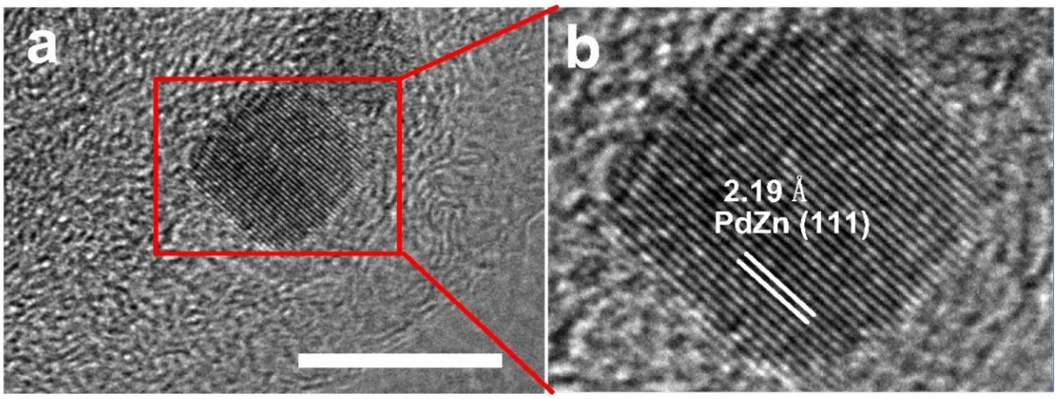

Supplementary Figure 8 | HR-TEM image of PdZn/NC@ZnO. a HR-TEM image of PdZn/NC@ZnO. Scale bar, 10 nm. **b** One PdZn NP on [111] zone axis.

The lattice spacing of 2.19 Å also agrees with interplane distances of (111) plane of the intermetallic PdZn.

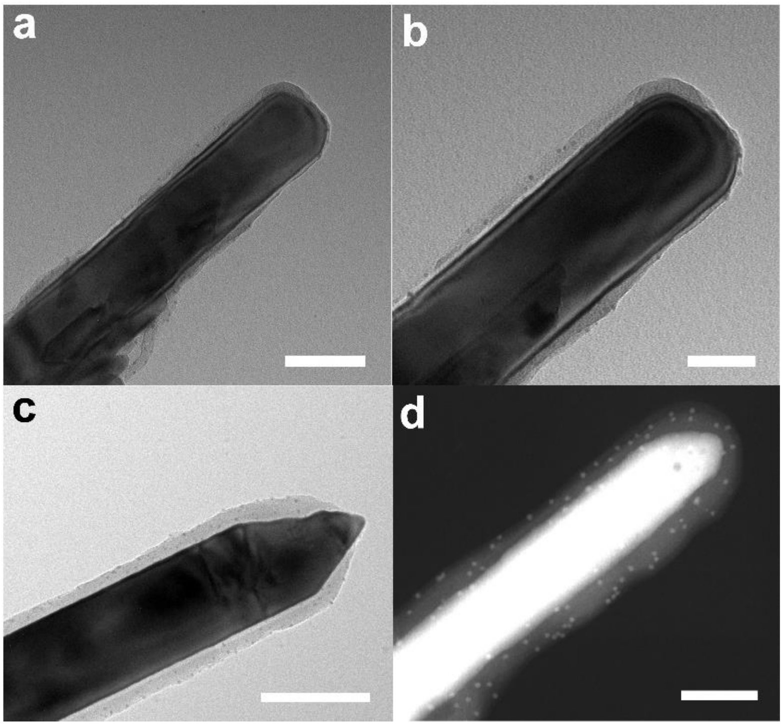

Supplementary Figure 9 | TEM and HR-TEM characterizations of the as-obtained Pd_n/NC@ZnO. a TEM image of Pd_n/NC@ZnO. Scale bar, 100 nm. **b** TEM image of Pd_n/NC@ZnO. Scale bar, 50 nm. **c** HR-TEM image of Pd_n/NC@ZnO. Scale bar, 100 nm. **d** STEM image of Pd_n/NC@ZnO. Scale bar, 50 nm.

From above images of the as-obtained Pd_n/NC@ZnO, it can be found that small-sized Pd nanoparticles are uniformly distributed on the NC@ZnO carrier.

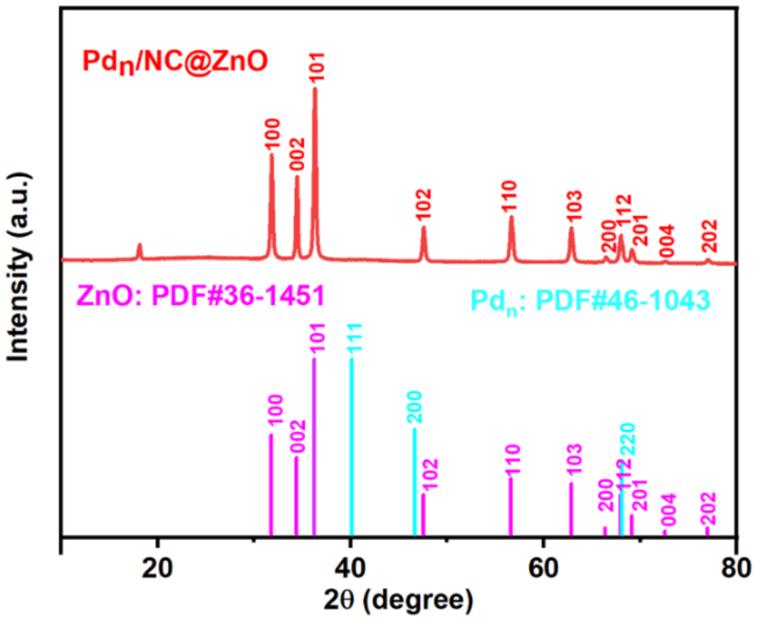

Supplementary Figure 10 | XRD pattern of Pd_n/NC@ZnO.

The diffraction peaks for Pd_n/NC@ZnO can match well with that of ZnO. No obvious peaks of Pd can be observed in the Pd_n/NC@ZnO diffraction patterns due to Pd nanoparticles being small enough.

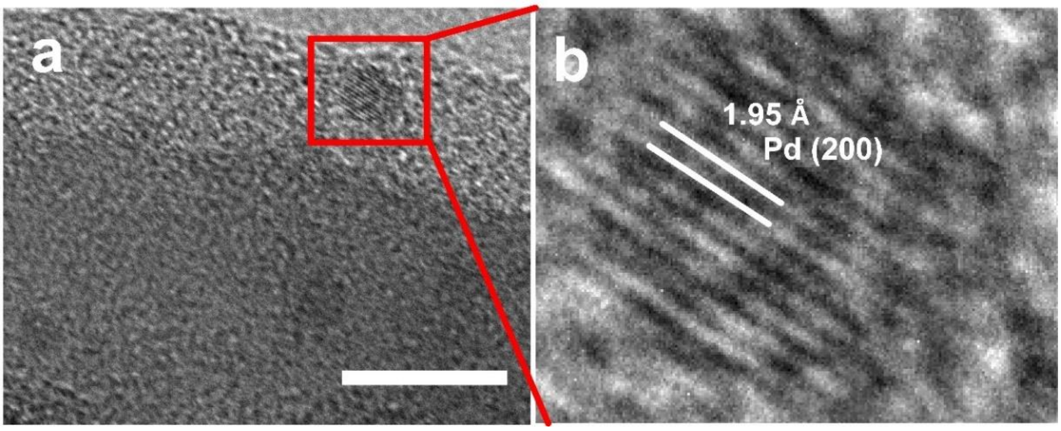

Supplementary Figure 11 | HR-TEM images of Pd_n/NC@ZnO. a HR-TEM overview image of Pd_n/NC@ZnO. Scale bar, 10 nm; **b** HR-TEM image of one Pd nanoparticle on [200] zone axis.

The lattice spacing of 1.95 Å also agrees with interplane distances of Pd (200) plane, proving the formed nanoparticles being Pd nanoparticles.

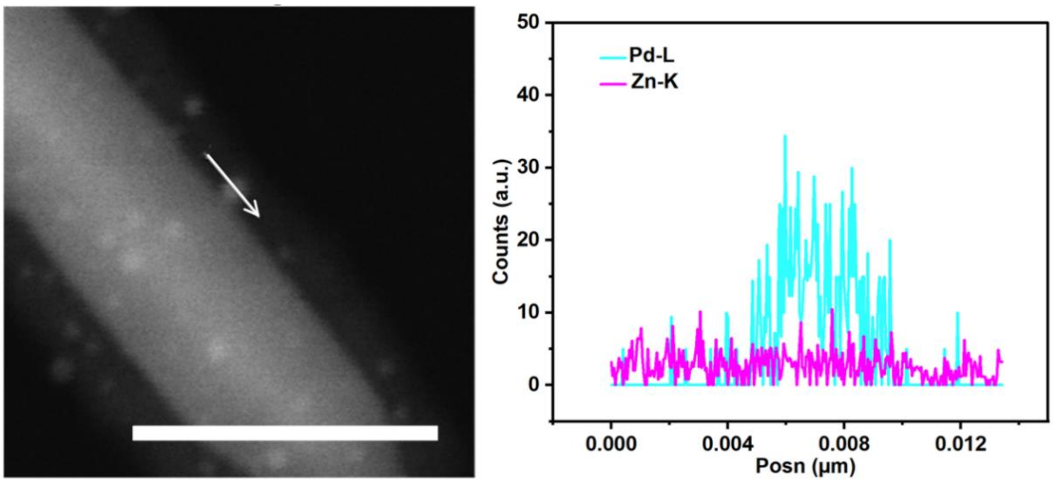

Supplementary Figure 12 | EDS line scanning profile across one nanoparticle in Pd_n/NC@ZnO. Scale bar, 50 nm.

It shows that there is only the signal of Pd but no signal of Zn, further indicating that the nanoparticle is composed of Pd.

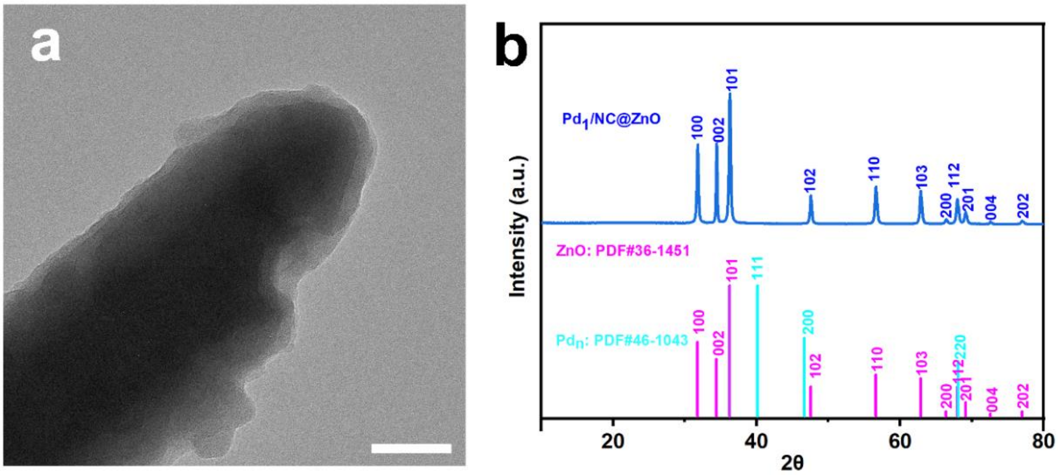

Supplementary Figure 13 | TEM image and XRD pattern of Pd_1/NC@ZnO. a TEM image of Pd_1/NC@ZnO. Scale bar, 50 nm; **b** XRD pattern of Pd_1/NC@ZnO.

It can be seen from the TEM image of Pd_1/NC@ZnO that no nanoparticles are observed on the NC@ZnO. The diffraction peaks for Pd_1/NC@ZnO can match well with that of ZnO. No obvious peaks of Pd can be observed in the Pd_1/NC@ZnO diffraction patterns, suggesting the presence of individual Pd sites.

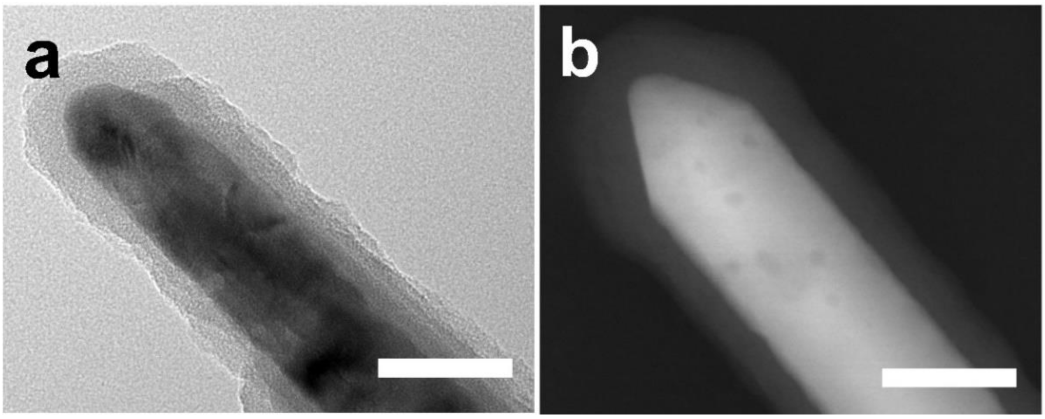

Supplementary Figure 14 | HR-TEM and STEM images of Pd_1/NC@ZnO. a HR-TEM image of Pd_1/NC@ZnO. Scale bar, 50 nm; **b** STEM image of Pd_1/NC@ZnO. Scale bar, 50 nm.

As can be seen from HR-TEM and STEM images of Pd_1/NC@ZnO, no nanoparticles can be observed on the NC@ZnO either, indicating Pd species existing as individual sites.

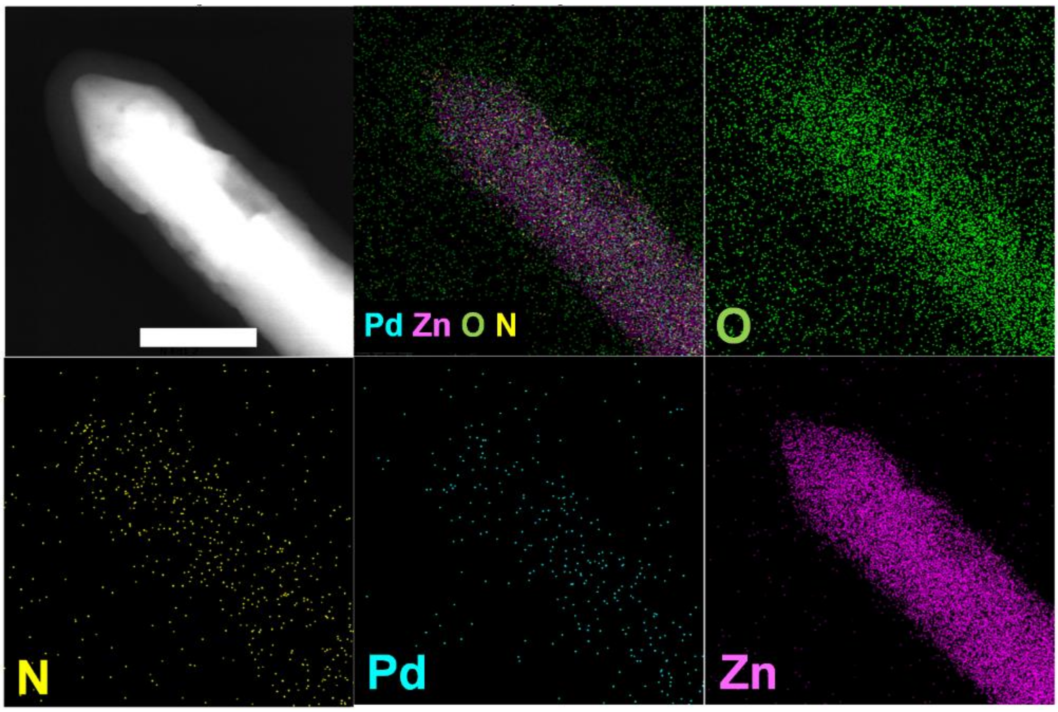

Supplementary Figure 15 | EDX elemental mappings of Pd_1/NC@ZnO. Scale bar, 100 nm.

It shows that Pd an N species distribute uniformly on the NC@ZnO.

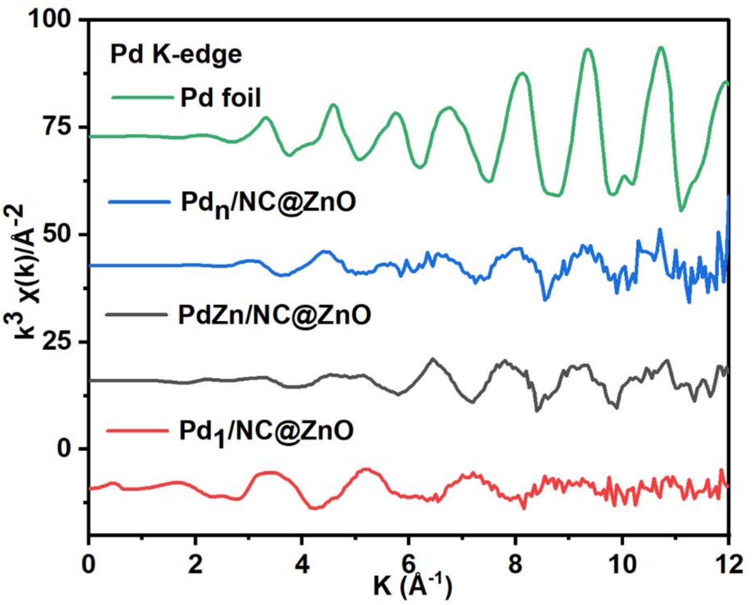

Supplementary Figure 16 | The EXAFS oscillation functions at the Pd K-edge of PdZn/NC@ZnO, Pd_n/NC@ZnO, Pd_1/NC@ZnO and Pd foil.

Through the EXAFS oscillation functions at the Pd K-edge of PdZn/NC@ZnO in comparison with Pd_n/NC@ZnO, Pd_1/NC@ZnO and Pd foil samples, the first nearest-coordination peak of PdZn/NC@ZnO displayed a slight shift in k space than that of Pd foil at Pd K-edge.

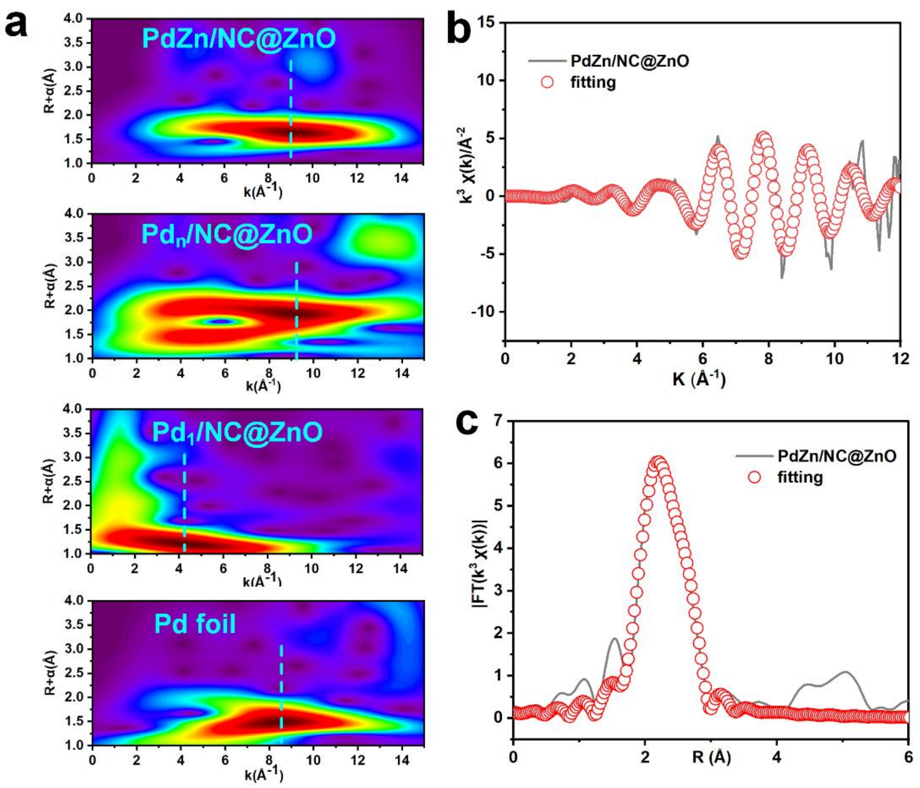

Supplementary Figure 17 | Wavelet transform (WT) and EXAFS curve fitting. a Wavelet transform (WT) of PdZn/NC@ZnO, Pd_n/NC@ZnO, Pd_1/NC@ZnO and Pd foil samples, respectively. **b, c** EXAFS curve fitting of PdZn/NC@ZnO.

Supplementary Table 1 | Pd K-edge EXAFS curves fitting parameters.

Sample	Shell	N	R (Å)	σ^2 ($\times10^{-3}$Å^2)	ΔE_0 (eV)	R factor
PdZn/NC@ZnO	Pd-Zn	5.0 ± 1.5	2.60 ± 0.05	12.3 ± 2.2	1.2 ± 2.9	0.02
	Pd-Pd	3.0 ± 1.3	2.79 ± 0.11	12.3 ± 2.2		

N is the coordination number N; R is interatomic distance (the bond length between central atoms and surrounding coordination atoms); σ^2 is Debye–Waller factor (a measure of thermal and static disorder in absorber-scatter distances); ΔE_0, is edge-energy shift (the difference between the zero inetic energy value of the sample and that of the theoretical model); R factor is used to value the goodness of the fitting. S_0^2 was fixed to 1.

Through the Wavelet transform (WT) of PdZn/NC@ZnO in comparison with Pd_n/NC@ZnO, Pd_1/NC@ZnO and Pd foil samples, the coordination configuration of Pd atom for PdZn/NC@ZnO is investigated by quantitative EXAFS curve fitting analysis. The best-fitting analyses clearly present a main peak originating from Pd-Zn first shell coordination. The coordination peak of Pd in PdZn/NC@ZnO locates at the smaller R value of 2.2 Å in comparison with that in Pd foil at 2.5 Å from the Pd-Pd contribution. The fitting analysis elucidates that this first shell peak is contributed from Pd-Zn (~2.6 Å) and longer Pd-Pd (~2.8 Å) bonds, coinciding with the atomic structure of intermetallic PdZn compound.

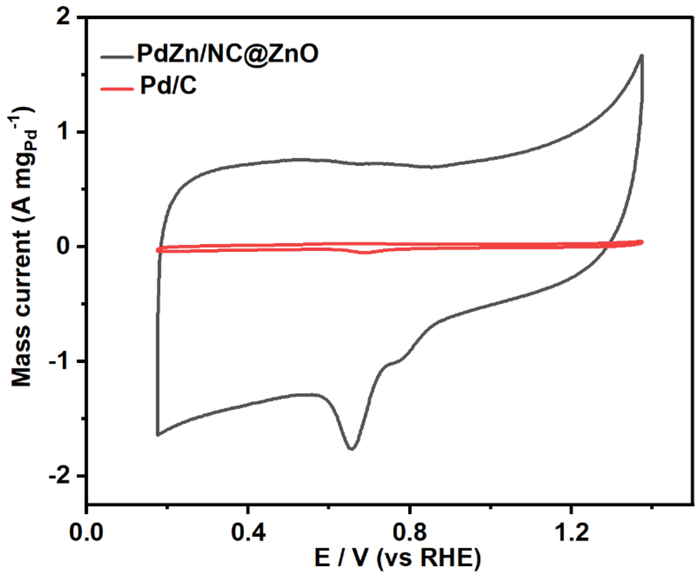

Supplementary Figure 18 | CV curves of commercial Pd/C and PdZn/NC@ZnO catalysts in 1.0 M KOH solution without ethanol.

CV curves of commercial Pd/C and PdZn/NC@ZnO catalysts measures at room temperature in N_2-purged 1.0 M KOH at a sweep rate of 0.05 V s^{-1}. The invariability of the curve during several cycles suggests that the surface of Pd and PdZn has reached a relatively stable state by the activation process. With the scans moving from 0.10 to 1.40 V (vs RHE), oxidation peaks appear due to the formation of surface Pd oxides. During the negative scans, the peaks at ≈ 0.7 V (vs RHE) correspond to the reduction of the oxides.

Supplementary Table 2 | EOR performance of PdZn/NC@ZnO catalyst and state-of-the-art Pd-based nanocatalysts from recent published works.

Catalyst	Mass Activity	Electrolyte	References
PdZn/NC@ZnO	18.14 A mg_{Pd}^{-1}	1.0 M KOH + 1.0 M ethanol	This work
PGM-HEA	~ 1.6 A $mg_{all\text{-}metals}^{-1}$	1.0 M KOH + 1.0 M ethanol	1
Au-Pd aerogel	8.4 A mg^{-1}	1.0 M KOH + 1.0 M ethanol	2
PVP-modified Au-Pd aerogels	9.75 A mg_{Pd}^{-1}	1.0 M KOH + 1.0 M ethanol	3
ultrathin Pd nanomeshes	5.40 A mg^{-1}	1.0 M KOH + 1.0 M ethanol	4
Au-Pd aerogel	8.45 A mg_{Pd}^{-1}	1.0 M KOH + 1.0 M ethanol	5
PdSn/SnOx	3.2 A mg^{-1}	1.0 M KOH + 1.0 M ethanol	6
c-Pd-Ni-P@a-Pd-Ni-P nanoplates	3.05 A mg_{Pd}^{-1}	1.0 M KOH + 1.0 M ethanol	7
Pd_2Sn:P/C	5.6 A mg_{Pd}^{-1}	0.5 M KOH + 0.5 M ethanol	8
Pd/NCB@NGS-2	2.70 A mg_{Pd}^{-1}	1.0 M KOH + 1.0 M ethanol	9
$Pd_{61}Pt_{22}Cu_{17}$TNRs/C	12.42 A mg_{Pd+Pt}^{-1}	1.0 M KOH + 1.0 M ethanol	10
Pd–Co_2P–PdSAs@GO	10.52 A mg_{Pd}^{-1}	1.0 M KOH + 1.0 M ethanol	11

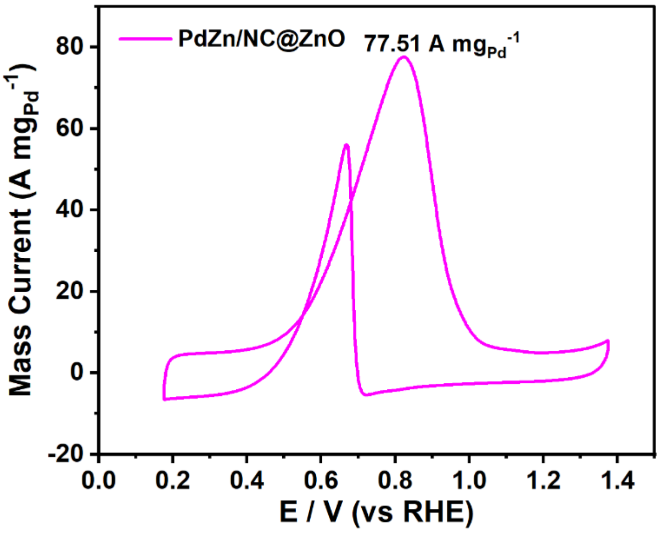

Supplementary Figure 19 | CV curve of PdZn/NC@ZnO based on surfaced Pd atoms on PdZn NPs in PdZn/NC@ZnO. The data is recorded in N_2-saturated 1.0 M KOH and 1.0 M C_2H_5OH at room temperature at scan rate of 50 mV s^{-1}.

According to the per surface atom calculation, the current density of PdZn/NC@ZnO for EOR is as high as 77.51 A mg_{Pd}^{-1}.

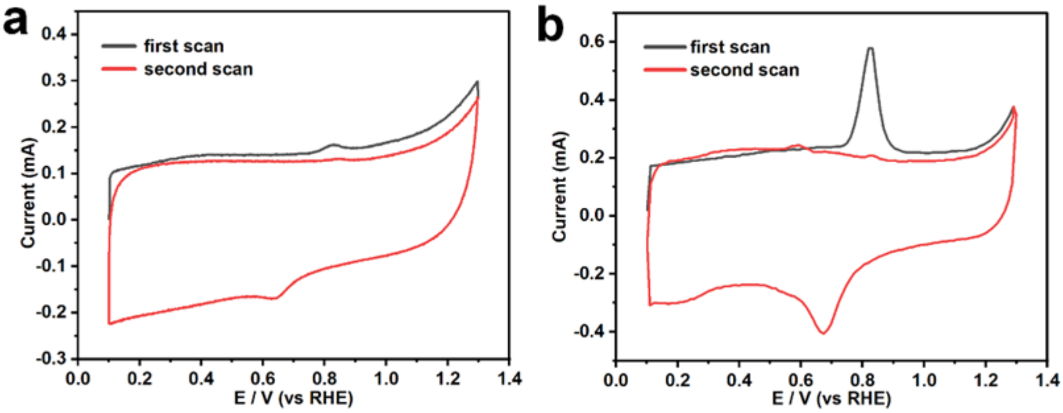

Supplementary Figure 20 | CO stripping plots for determining ECSA. a CO stripping plot of PdZn/NC@ZnO between 0.10 and 1.40 V vs RHE in 1.0 M KOH at a sweep rate of 0.02 V s^{-1}. **b** CO stripping plot of commercial Pd/C between 0.10 and 1.40 V vs RHE in 1.0 M KOH at a sweep rate of 0.02 V s^{-1}.

As shown in the typical CO-stripping plots of the PdZn/NC@ZnO and commercial Pd/C catalysts, the red line represents the full oxidation of CO in the first cycle and the blue line represents typical CV curves in 1.0 M KOH solution without any CO adsorption. PdZn/NC@ZnO and commercial Pd/C catalysts exhibit an anodic wave centered at around 0.80 V versus RHE, which corresponds to the electrooxidation of the adsorbed CO on the catalyst surface. In comparison with the commercial Pd/C, the PdZn/NC@ZnO catalyst shows a much smaller CO stripping peak.

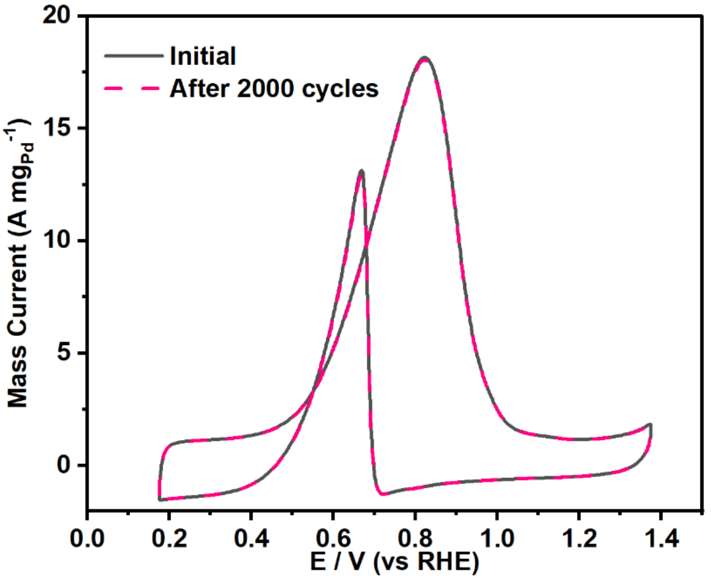

Supplementary Figure 21 | Electrochemical stability of PdZn/NC@ZnO catalyst.

CV curves before and after 2000 potential cycles are measured between 0.10 and 1.40 V vs RHE in 1.0 M KOH + 1.0 M CH_3CH_2OH at a sweep rate of 0.05 V s^{-1}. The current is normalized by the loading amount of Pd on the electrode. The stability of PdZn/NC@ZnO in the EOR is examined by continuous CV scans in the electrolyte with ethanol. It is obvious that there is almost no change in the CV curves after 2000 cycles, indicating the good catalytic stability of PdZn/NC@ZnO.

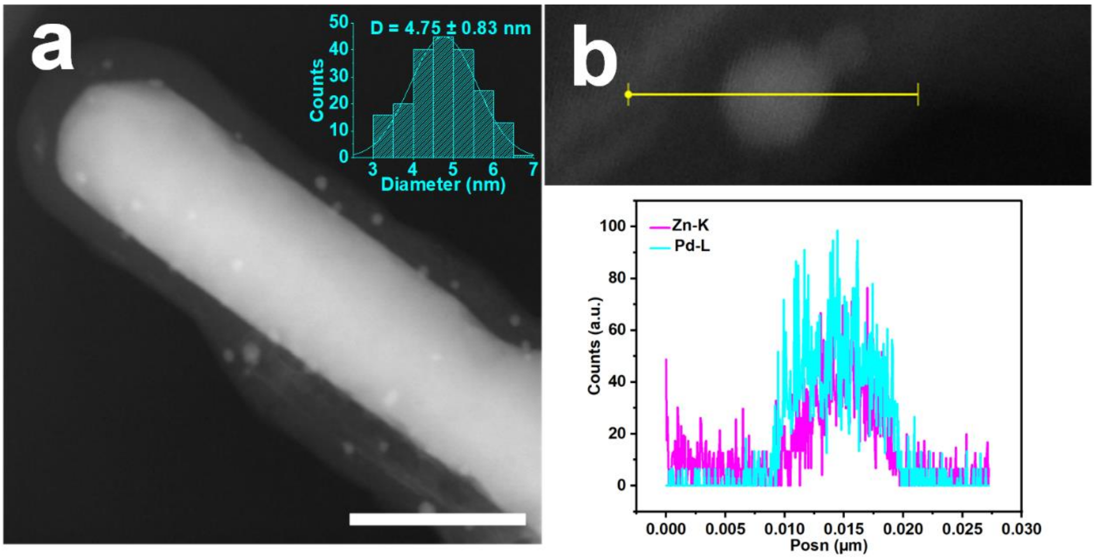

Supplementary Figure 22 | STEM image and EDS line scanning. **a** STEM image of PdZn/NC@ZnO after the stability test, the inset in (a) is the size distribution histogram of PdZn NPs. Scale bar, 100 nm. **b** EDS line scanning of PdZn/NC@ZnO after the stability test.

The catalyst after potential cycles shows no observable changes in the morphology and composition, proving the desired stability of PdZn/NC@ZnO for EOR.

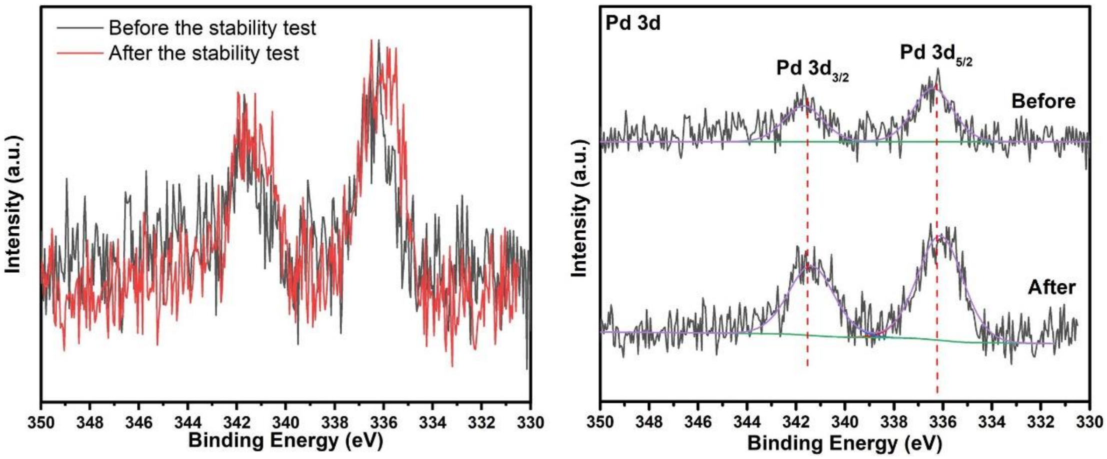

Supplementary Figure 23 | Pd *3d* XPS spectra of PdZn/NC@ZnO before and after the stability test.

After the stability test, it can be evidenced from XPS spectra that Pd *3d* signals of PdZn/NC@ZnO keep unchanged, revealing the stable structure of Pd-Zn dual sites.

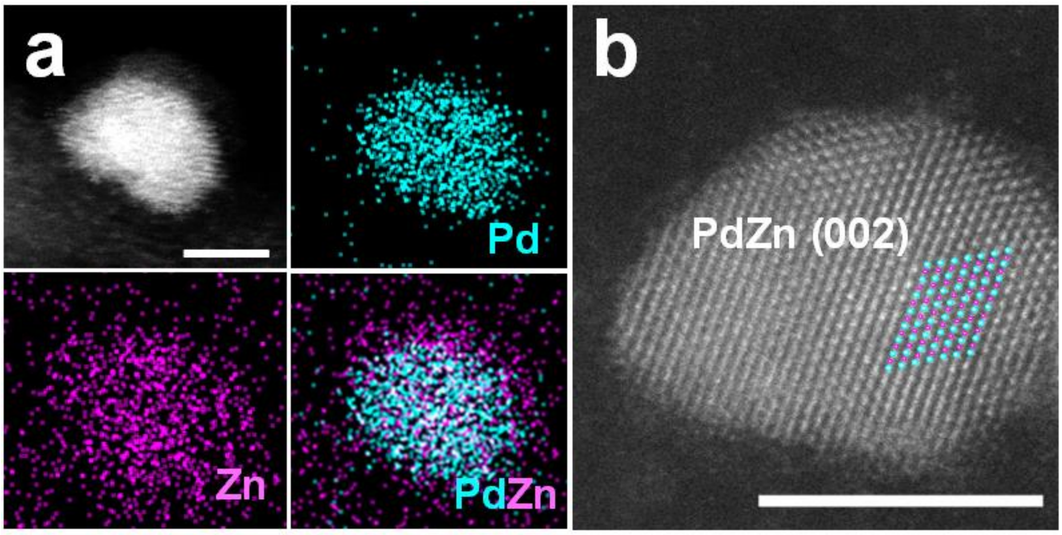

Supplementary Figure 24 | AC HAADF-STEM characterization of PdZn/NC@ZnO after the stability test. a AC HAADF-STEM image of one PdZn nanoparticle in PdZn/NC@ZnO after the stability test. Scale bar, 2.5 nm. **b** AC HAADF-STEM elemental mappings of one PdZn nanoparticle in PdZn/NC@ZnO after the stability test. Scale bar, 5 nm.

Atomic-resolution EDS elemental mapping analysis manifests that Pd and Zn homogeneously disperse over the entire nanoparticle (Supplementary Fig. 24a). The typical AC-HAADF STEM image of one nanoparticle exhibits clear bright dots, within which the heavier Pd atoms (bright) can be distinguished from Zn atoms (darker) (Supplementary Fig. 24b). More importantly, the ordering of these bright dots accords with the atomic arrangement of (002) plane in the intermetallic PdZn (P4/mmm) crystal structure. These results testify the intermetallic nature of PdZn NPs with uniform Pd-Zn dual sites in PdZn/NC@ZnO after the stability test.

Supplementary Table 3 | ICP-OES detections of PdZn/NC@ZnO and solution after the stability test.

Element	PdZn/NC@ZnO (wt%)	Solution (μg mL^{-1})
Pd	0.3	< 100
Zn	-	< 20

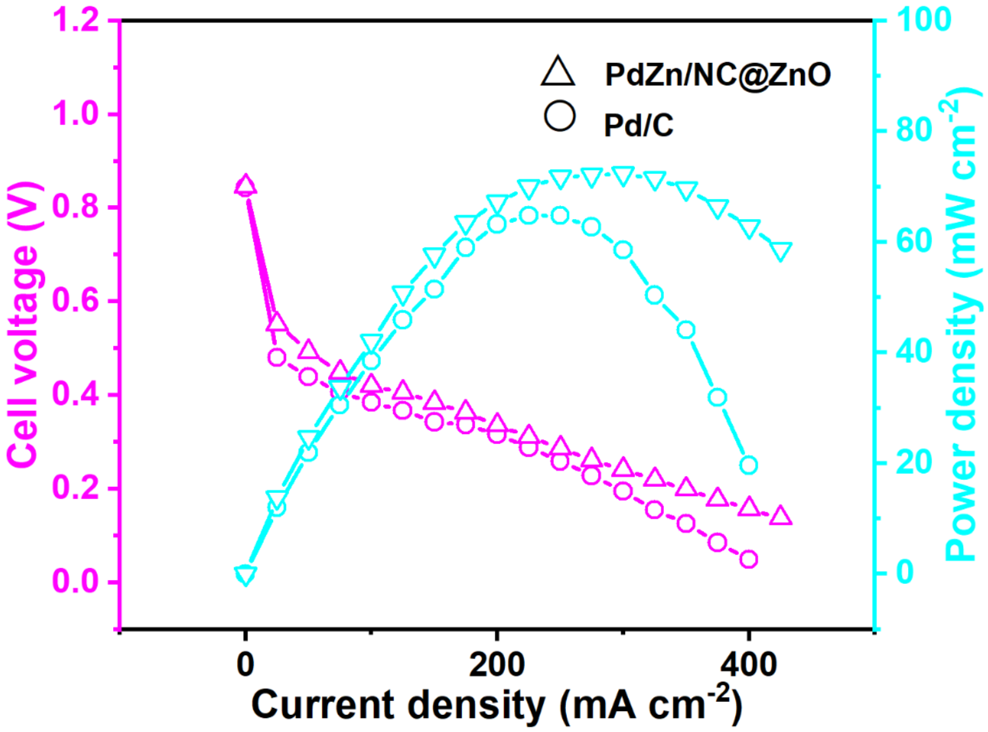

Supplementary Figure 25 | The polarization and power density curves for DEFC. PdZn/NC@ZnO (0.8 mg cm^{-2} total catalyst loading) and Pd/C (1.0 mg cm^{-2} total catalyst loading) are used as anode catalysts respectively. All experiments are done using 1.0 M ethanol in 1.0 M KOH as a fuel feed. The fuel cell is conditioned at 50 °C with 2.0 mL min^{-1} fuel and 100 sccm O_2. Electrolyte: Nafion212 membrane.

As can be seen from the DEFC performance of PdZn/NC@ZnO and Pd/C, PdZn/NC@ZnO also displays the better performance than Pd/C under the real operational conditions of DEFC.

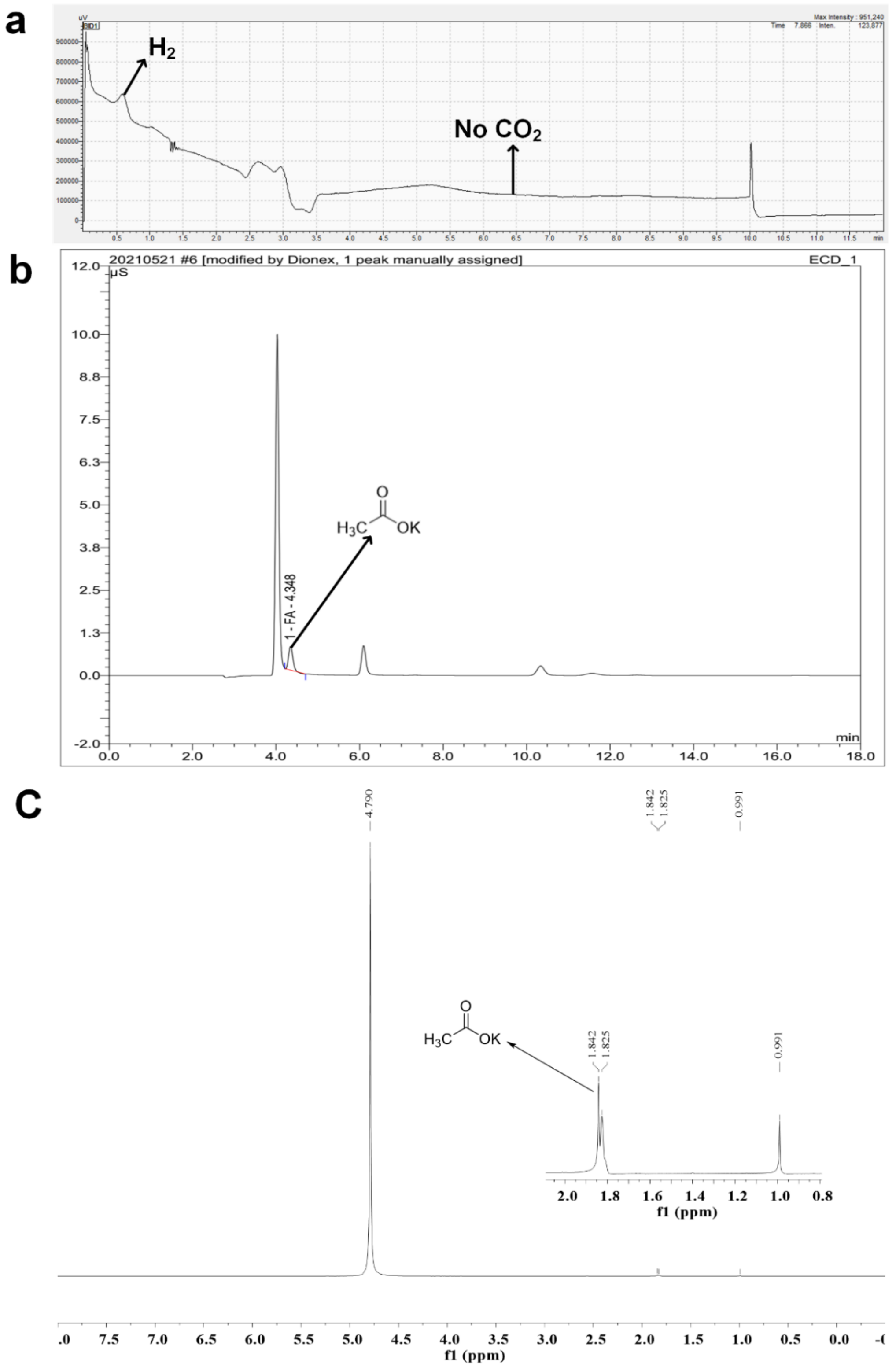

Supplementary Figure 26 | Detections on the products in gas and liquid phase of EOR over PdZn/NC@ZnO. a On-line GC spectrum of the gas phase in EOR. **b** IC spectrum of the liquid phase in EOR **c** ^{1}H NMR spectrum of the liquid phase in EOR.

According to the on-line GC results, the electrooxidation of ethanol over PdZn/NC@ZnO yield H_2 only without CO_2 in the gas phase. According to the IC and ^{1}H NMR results, acetic acid is formed in the liquid phase of EOR.

Supplementary Table 4 | Surface energy of different crystal planes.

Surface	Surface energy (J m^{-2})
PdZn(001)	2.13
PdZn(100)	2.07
PdZn(110)	1.88

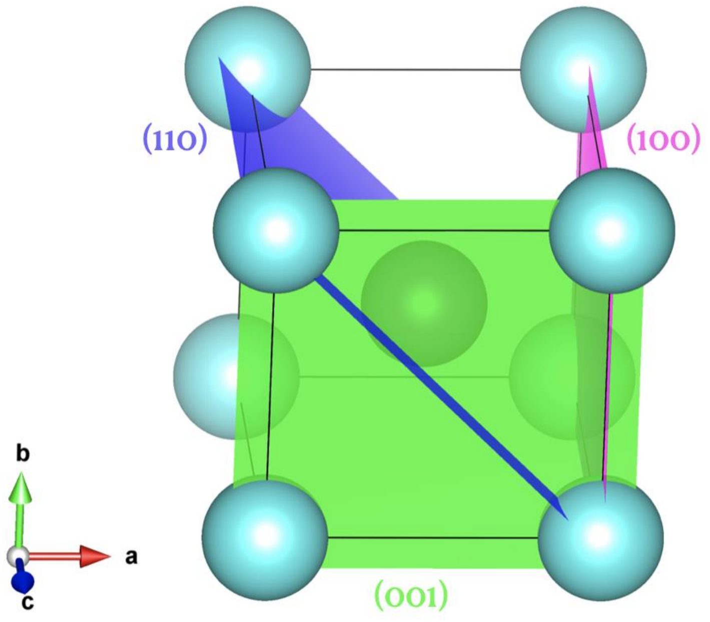

Supplementary Figure 27 | Geometry of intermetallic PdZn primitive cell as well as the selected (100), (001) and (110) surfaces.

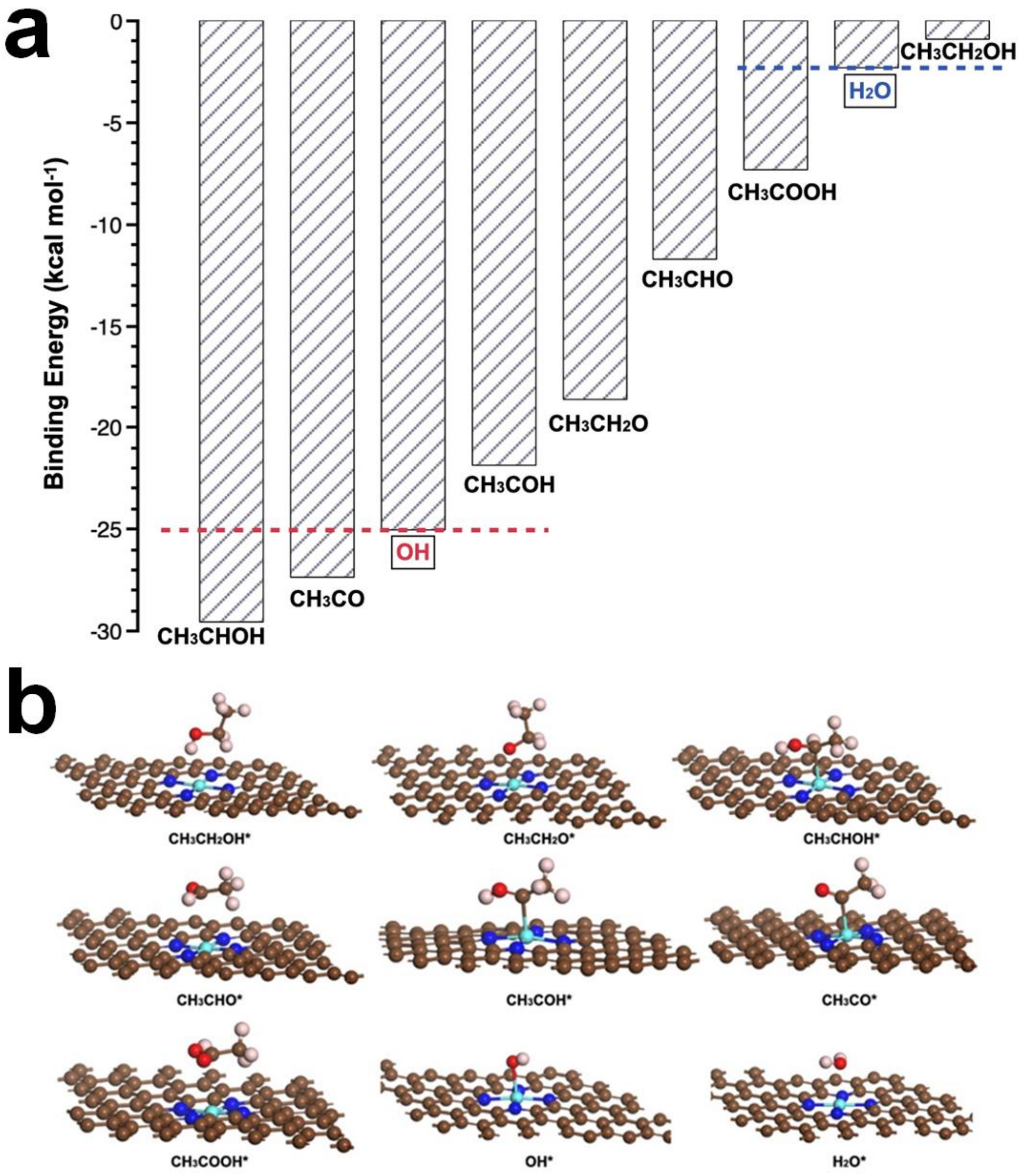

Supplementary Figure 28 | DFT calculations on the EOR over individual Pd sites. a Binding energy of adsorbates on modulated individual Pd sites. **b** Adsorption configurations of adsorbates on modulated individual Pd sites.

We calculated the binding energies of all the intermediates considered. The binding energies and adsorption configurations are shown in Supplementary Fig. 28. We can find that intermediates with unsaturated carbon atoms, such as CH_3CHOH, CH_3CO and CH_3COH bind stronger on the individual Pd sites than the intermediates with saturate carbon atoms (CH_3CH_2O, CH_3CHO, CH_3COOH and CH_3CH_2OH). Binding strength between CH_3CH_2OH and individual Pd site is quite weak, even weaker than it for water molecule. Furthermore, binding energy for OH is very strong. Hence the ER mechanism should be triggered by the adsorbed OH and the bombarding EtOH molecule.

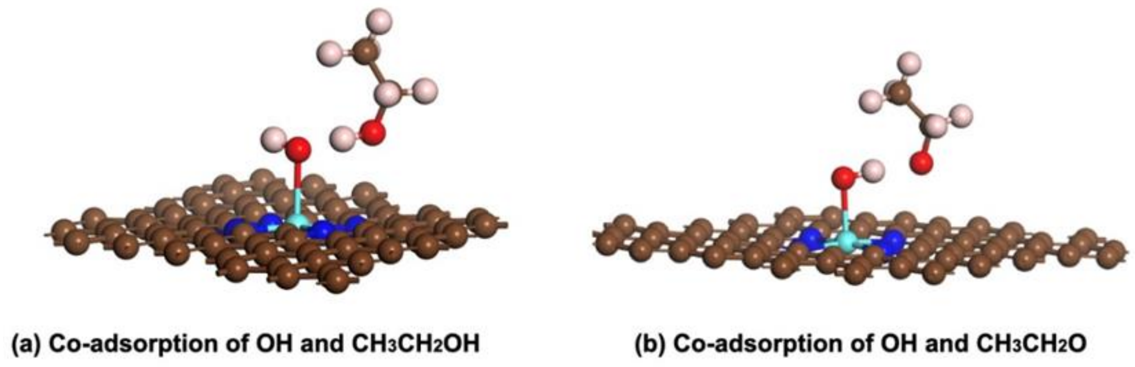

Supplementary Figure 29 | DFT calculations on the EOR over individual Pd sites. The co-adsorption configurations of OH…CH_3CH_2OH (**a**) and OH…CH_3CH_2O (**b**) on individual Pd sites.

Our theoretical studies suggested that for individual Pd sites the EOR process would be interrupted due to the exclusive hydrogen bond when CH_3CH_2O is formed.

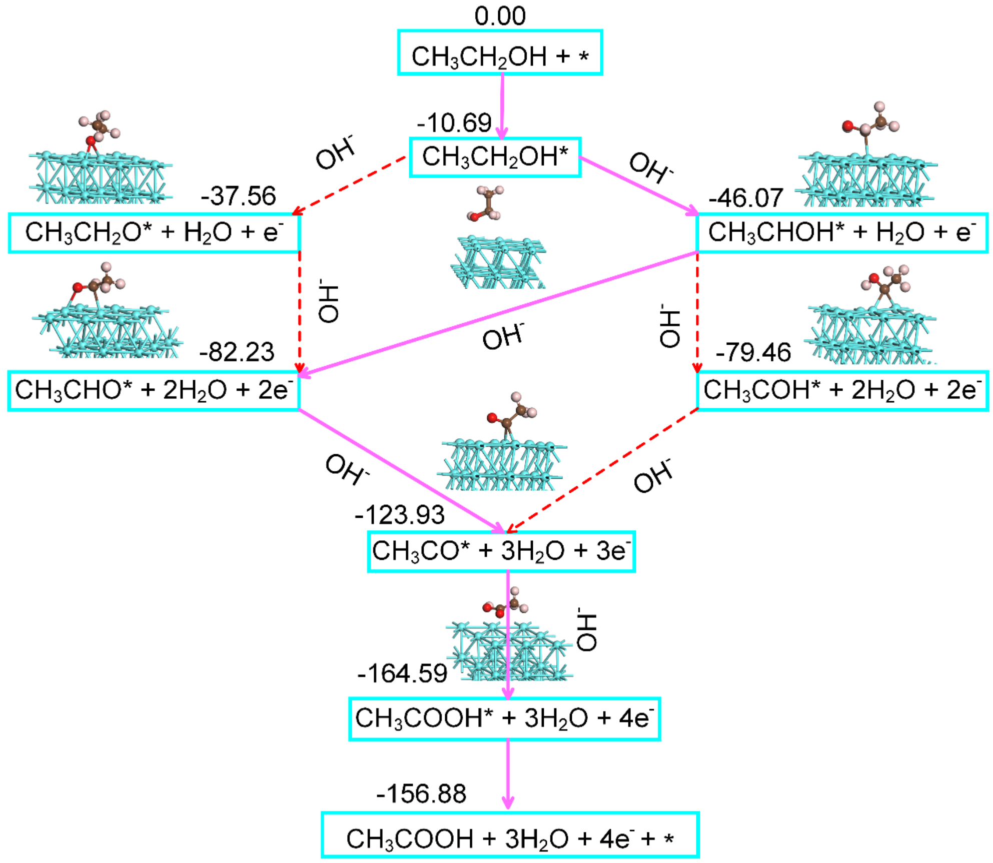

Supplementary Figure 30 | DFT-calculated reaction mechanisms of EOR on Pd-Pd sites. For the reactions on Pd-Pd sites associated structure model adsorbed with the reactive species from different states. DFT-calculated EOR free-energies on Pd-Pd sites at pH = 14, U= 0.82 V with

respect to the RHE. The purple solid line arrow represents the easy reaction path, and the red dotted line arrow represents the more difficult reaction path.

EOR on Pd-Pd sites undergoes an indirect pathway, i.e., CH_3CH_2OH → CH_3CHOH^* → CH_3CHO^* → CH_3CO^*, followed by CH_3CO interaction with surface hydroxyl (OH^*) to form CH_3COOH, agreeing with previous study[8].

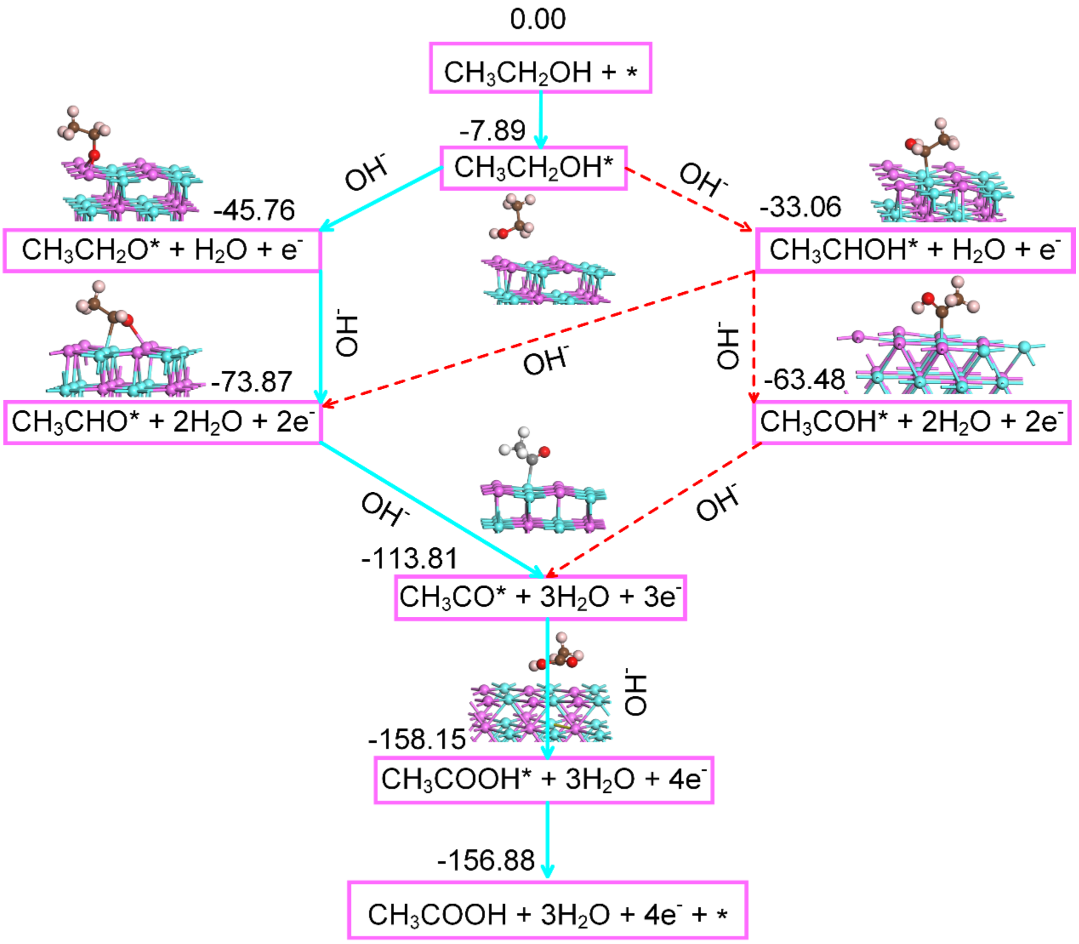

Supplementary Figure 31 | DFT-calculated reaction mechanisms of EOR on Pd-Zn dual sites. For the reactions on Pd-Zn dual sites associated structure model adsorbed with the reactive species from different states. DFT-calculated EOR free-energies on Pd-Zn dual sites at pH = 14, U = 0.82 V with respect to the RHE. The green solid line arrow represents the easy reaction path, and the red dotted line arrow represents the more difficult reaction path.

EOR on Pd-Zn dual sites undergoes an indirect pathway, i.e., CH_3CH_2OH → $CH_3CH_2O^*$ → CH_3CHO^* → CH_3CO^*, followed by CH_3CO interaction with surface hydroxyl (OH^*) to form CH_3COOH.

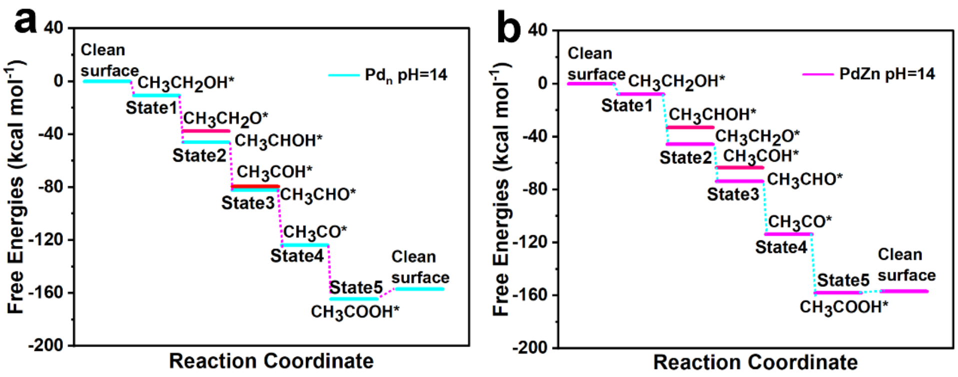

Supplementary Figure 32 | DFT calculated reaction pathway of EOR on Pd-Pd sites and Pd-Zn dual sites. The free energies profiles of EOR reactions over both Pd-Pd sites on Pd (111) (**a**) and Pd-Zn dual sites on PdZn (110) (**b**) surfaces at pH = 14, U= 0.82 V with respect to the RHE.

Because of the different reaction paths and the low energy required for acetic acid removal on the Pd-Zn dual sites, the Pd-Zn dual sites are more conducive to the oxidation of ethanol.

References.

1. Wu D. *et al.* Platinum-group-metal high-entropy-alloy nanoparticles. *J. Am. Chem. Soc.* **142**, 13833-13838 (2020).
2. Du R. *et al.* Freeze-thaw-promoted fabrication of clean and hierarchically structured noble-metal aerogels for electrocatalysis and photoelectrocatalysis. *Angew. Chem. Int. Ed.* **59**, 8293-8300 (2020).
3. Fan X. *et al.* Promoting the electrocatalytic performance of noble metal aerogels by ligand-directed modulation. *Angew. Chem. Int. Ed*. **59**, 5706-5711 (2020).
4. Ge J. *et al.* ultrathin palladium nanomesh for electrocatalysis. *Angew. Chem. Int. Ed.* **57**, 3435-3438 (2018).
5. Du R. *et al.* Unveiling reductant chemistry in fabricating noble metal aerogels for superior oxygen evolution and ethanol oxidation. *Nat. Commun.* **11**, 1590 (2020).
6. Gao Q. *et al.* Monodisperse PdSn/SnOx core/shell nanoparticles with superior electrocatalytic ethanol oxidation performance. *J. Mater. Chem. A.* **8**, 20931-20938 (2020).
7. Yin P. *et al.* Synthesis of palladium-based crystalline@amorphous core-shell nanoplates for highly efficient ethanol oxidation. *Adv. Mater.* **32**, e2000482 (2020).
8. Yu X*, et al.* Phosphorous incorporation in Pd_2Sn alloys for electrocatalytic ethanol oxidation. *Nano Energy* **77**, 105116 (2020).
9. Li S. *et al*. Engineering three-dimensional nitrogen-doped carbon black embedding nitrogen-doped graphene anchoring ultrafine surface-clean Pd nanoparticles as efficient ethanol oxidation electrocatalyst. *Appl. Catal. B.* **280**, 119464 (2021).
10. Wang W. *et al*. Edge enrichment of ultrathin 2D PdPtCu trimetallic nanostructures effectuates top-ranked ethanol electrooxidation. *Nano Lett.* **20**, 5458-5464 (2020).
11. Tiwari J. *et al*. Remarkably enhanced catalytic activity by the synergistic effect of palladium single atoms and palladium–cobalt phosphide nanoparticles. *Nano Energy* **78**, 105166 (2020).
